# Effect of curcumin nanoparticles on proliferation and migration of mouse airway smooth muscle cells and airway inflammatory infiltration

**DOI:** 10.3389/fphar.2024.1344333

**Published:** 2024-04-19

**Authors:** Yucong Ma, Suping Ye, Kunpeng Sun, Yue Gu

**Affiliations:** ^1^ Department of Pediatric Respiration, Children’s Medical Center, The First Affiliated Hospital of Jilin University, Changchun, Jilin, China; ^2^ Department of Reparatory and Critical Care Medicine, The First Affiliated Hospital of Jilin University, Changchun, Jilin, China

**Keywords:** curcumin, nanoparticles, proliferation, migration, airway smooth muscle cells, airway inflammatory infiltration

## Abstract

Curcumin (CUR) possesses the capability to inhibit various inflammatory factors, exert anti-inflammatory effects, and alleviate asthma attacks; however, its hydrophobicity and instability significantly impede its clinical application. In this study, we synthesized CUR-loaded nanoparticles (CUR-NPs) and evaluated their impact on the proliferation, migration, and inflammatory infiltration of mouse airway smooth muscle cells (ASMCs), while investigating their underlying mechanisms. To achieve this objective, ASMCs were isolated from BALB/c mice and subjected to TGF-β1-induced cell proliferation and migration. Our findings demonstrate that CUR-NPs effectively regulate the release of CUR within cells with superior intracellular uptake compared to free CUR. The CCK-8 assay results indicate that the blank carrier does not exhibit any cytotoxic effects on cells, thus rendering the impact of the carrier itself negligible. The TGF-β1 group exhibited a significant increase in cell proliferation, whereas treatment with CUR-NPs significantly suppressed TGF-β1-induced cell proliferation. The findings from both the cell scratch assay and transwell assay demonstrated that TGF-β1 substantially enhanced cell migration, while CUR-NPs treatment effectively attenuated TGF-β1-induced cell migration. The Western blot analysis demonstrated a substantial increase in the expression levels of TGF-β1, p-STAT3, and CTGF in ASMCs following treatment with TGF-β1 when compared to the control group. Nevertheless, this effect was effectively counteracted upon administration of CUR-NPs. Furthermore, an asthma mouse model was successfully established and CUR-NPs were administered through tail vein injection. The serum levels of TGF-β1 and the expression levels of TGF-β1, p-STAT3, and CTGF proteins in the lung tissue of mice in the model group exhibited significant increases compared to those in the control group. However, CUR-NPs treatment effectively attenuated this change. Our research findings suggest that CUR-NPs possess inhibitory effects on ASMC proliferation, migration, and inflammatory infiltration by suppressing activation of the TGF-β1/p-STAT3/CTGF signaling pathway, thereby facilitating inhibition of airway remodeling.

## Introduction

Bronchial asthma (asthma) is a chronic inflammatory disease characterized by airway multicellularity and cellular components (Elkomy et al., 2022; Habib et al., 2022). With the continuous advancement of industrial society and the escalating environmental pollution, the prevalence of asthma has been progressively increasing, posing a significant threat to human health. Prolonged recurrence of asthma can lead to varying degrees of airway remodeling in affected individuals (Varricchi et al., 2022). Airway remodeling can result in alterations to airway function and organ properties, which is a crucial pathological factor leading to irreversible airway obstruction and hyperresponsiveness (Hsieh et al., 2023; Khalfaoui and Pabelick, 2023). Additionally, airway remodeling can exacerbate airway inflammation or secondary infection in asthma patients, and in severe cases, it may lead to increased mortality among those with asthma (Joseph and Tatler, 2022). Currently, the prevailing clinical interventions for asthma encompass glucocorticoids, β2 receptor agonists, aminophylline, and other pharmaceuticals that effectively alleviate airway inflammation in the majority of asthma patients (Zhao et al., 2019; Aralihond et al., 2020; Ramos-Ramirez and Tliba, 2022). Nevertheless, prolonged and repetitive usage of these treatments may engender a range of adverse effects, imposing significant psychological burdens on patients. Consequently, exploring novel therapeutic agents as substitutes or adjuncts to the aforementioned drugs has emerged as a pivotal research avenue in asthma management.

Studies have demonstrated that airway remodeling primarily arises from structural alterations in the airway wall induced by chronic inflammatory stimulation (Joseph and Tatler, 2022). These changes are predominantly characterized by proliferation and hypertrophy of airway smooth muscle cells, deposition of extracellular matrix, thickening of the basal membrane, infiltration of inflammatory cells, and glandular hypertrophy (Huang and Qiu, 2022). Airway smooth muscle cells (ASMCs) constitute the parenchymal component of airway smooth muscle and play a crucial role in airway remodeling in asthma. They possess pivotal functions including proliferation, migration, and secretion, thereby releasing pro-inflammatory, anti-inflammatory mediators as well as immunomodulatory factors to regulate airway inflammation (Salter et al., 2017). The aberrant proliferation and migration of airway smooth muscle cells play crucial roles in the pathogenesis of airway remodeling (Kume et al., 2023). Therefore, it is imperative to investigate novel targets and therapeutics for asthma treatment by modulating the proliferation and migration of ASMCs.

TGF-β, a cytokine secreted by immune cells and epithelial cells, plays a crucial role in regulating cell growth and differentiation, as well as in the modulation of airway remodeling in asthma (Devan et al., 2022). The expression of TGF-β1 is significantly upregulated in the lung tissues of mice or rats with asthma-induced airway remodeling (Liu et al., 2015). Activation of TGF-β1 receptors can initiate downstream signaling pathways, such as the STAT3 pathway (Xu et al., 2014), leading to increased CTGF protein expression (Wang et al., 2018). Studies have also demonstrated that TGF-β1 can activate the STAT3 signaling pathway in hepatocytes and induce CTGF release (Makino et al., 2023). It is evident that the activation of TGF-β1 and CTGF through the STAT3 signaling pathway is associated with cell proliferation and migration, thereby influencing airway remodeling.

With the advent of novel medical experimental techniques, significant advancements have been achieved in experimental research on airway remodeling prevention and treatment using traditional Chinese medicine (Zhou et al., 2022). Traditional Chinese medicine exhibits distinctive characteristics such as multi-pathway, multi-link, and multi-target effects, rendering it highly promising for the prevention and treatment of airway remodeling compared to Western medicine. Curcumin (CUR), a polyphenolic compound derived from the roots and stems of the curcuma plant (Gong et al., 2021; Jabbar et al., 2023), exhibits potent anti-inflammatory and antioxidant properties (Peng et al., 2021). Notably, CUR demonstrates robust free radical scavenging activity when compared to natural extracts like anthocyanins and β-carotene (Sadatsharifi and Purgel, 2021), suggesting its superior antioxidant efficacy over vitamins. In addition to scavenging reactive oxygen species and reactive nitrogen, CUR also elicits the upregulation of antioxidant enzymes (Guo et al., 2020). Extensive research has been conducted on its efficacy in various inflammatory diseases such as allergic asthma, acute respiratory distress syndrome, and acute lung injury (Avasarala et al., 2013; Chong et al., 2014; Li K X et al., 2022). However, the limited solubility of CUR in water poses a challenge to its stability and effective maintenance of therapeutic blood concentrations (Nasery et al., 2020). Nanotechnology offers a promising solution to address these issues by utilizing suitable carriers for CUR delivery, which can prevent degradation, enhance intracellular accumulation, and improve bioavailability. In recent years, nanoparticles have gained significant attention as drug delivery systems for hydrophobic drugs due to their ability to prolong systemic circulation and thereby enhance efficiency and bioavailability (Hassani et al., 2020; Nasery et al., 2020; Li H T et al., 2022).

In this study, we aimed to fabricate CUR-loaded nanoparticles (CUR-NPs) using an amphiphilic copolymer PEG-PDLLA for the purpose of safeguarding CUR against degradation and enhancing its intracellular accumulation. Subsequently, mouse ASMCs were isolated from BALB/c mice and stimulated with transforming growth factor-β1 (TGF-β1) to induce cell proliferation and migration. The cellular activity was assessed using the cell counting kit-8 (CCK-8) method, while the cell migration ratio was determined through both cell scratch and transwell assays. Additionally, the protein expression levels of TGF-β1, total-signaling and the transcriptional activation factor 3 (STAT3), p-STAT3, and connective tissue growth factor (CTGF) in the cells were analyzed via Western blotting. Additionally, an asthma mouse model was established to quantify the levels of TGF-β1 in serum using enzyme-linked immunosorbent assay (ELISA), and evaluate the expression levels of TGF-β1, total-STAT3, p-STAT3, and CTGF proteins in lung tissue through Western blot analysis. The objective was to investigate the impact of CUR-NPs on the proliferation, migration, and inflammatory infiltration of ASMCs while exploring their mechanistic association with the TGF-β1/p-STAT3/CTGF signaling pathway.

## Experimental methods

### Chemical materials

The CUR was obtained from Shanghai Aladdin Reagent Co., Ltd. PEG-PDLLA (PEG, 5 kDa; PDLLA, 10 kDa) was obtained from Advanced Polymer Materials Inc. (Canada). The Bicinchoninic acid (BCA) protein quantification kit, hypersensitive chemiluminescence solution, and animal protein extraction reagents were all purchased from Thermo Fisher Scientific in the United States. The fetal bovine serum, 0.25% trypsin solution, penicillin-streptomycin dual antibody, and type II collagenase were all procured from Gibco Company in the United States. The cell culture medium was obtained from HyClone Company in the United States. Monoclonal antibodies such as TGF-β1, total-STAT3, p-STAT3, CTGF, β-actin and horseradish peroxidase-labeled goat anti-rabbit immunoglobulin were all purchased from Cell Signaling Technology (USA). Hybond C film was acquired from Pierce Company (USA). The CCK-8 kit and all other reagents were sourced from Sigma Aldrich (USA).

### Preparation and characterization of CUR-NPs

The CUR-NPs were prepared according to a previously described method (Chen M et al., 2018). In summary, a solution of CUR (1 mg) and PEG-PDLLA (9 mg) in tetrahydrofuran (4 mL) was added dropwise to 10 mL of distilled water with stirring. The resulting mixture was then dialyzed against water at room temperature for 3 days to form nanoparticles, which were subsequently lyophilized for further use. To determine the drug loading capacity (DL) and loading efficiency (LE) of nanoparticles, the nanoparticles were initially dispersed in methanol using ultrasonic method, followed by quantification of released small molecule CUR through high-performance liquid chromatography (Shimadzu LC-20AD, Japan). Determine the drug loading capacity (DL) and loading efficiency (LE) of CUR-NPs by employing the following formula:

$$\text{DL}\left(\%\right)=\left(M_{0}/M\right)\times 100\%$$ (1)

$$\text{LE}\left(\%\right)=\left(M_{0}/M_{1}\right)\times 100\%$$ (2)

Among them, M_0_ represents the mass of CUR encapsulated within the nanoparticles, M_1_ denotes the mass of CUR alone, and M signifies the mass of the nanoparticles.

Examine the morphological characteristics of nanoparticles using transmission electron microscopy (TEM, Tecnai G2-20). Determine the size distribution of nanoparticles utilizing a dynamic light scattering instrument equipped with a vertically polarized He Ne laser (Wyatt Technology, USA). Assess the potential of CUR-NPs employing a Nano-ZS instrument (Malvern, UK).

### *In Vitro* release

Take 2 mL of PBS with a pH of 7.4, containing tween-80 at a concentration of 1.0wt%. Suspend CUR-NPs in the PBS solution and transfer the mixture into a dialysis bag with a molecular weight cut-off value of 3.5 kDa to seal it. Submerge the dialysis bag in an 8 mL vial filled with PBS and shake the vial at 37^o^C. At the designated time point, withdraw 1 mL of culture medium from the vial and replace it with an equal volume of fresh buffer solution. Measure the absorbance at a wavelength of 427 nm using UV-vis analysis to quantify CUR release.

### Extraction and culture of mouse ASMCs

The mice were anesthetized and euthanized, followed by separation of the cervical trachea and lung tissues. The tissues were washed with PBS under a microscope, and other tissues were removed. Tissue blocks measuring 2–3 mm in size were cut and placed in sterile culture bottles for cultivation at 37^o^C for 4–6 h. Fresh culture medium was added for further cultivation upon observation of good adhesion. The supernatant was removed, and fresh culture medium was added to continue cultivation. The cells are cultured until they reach a density of approximately 90%. Subsequently, the supernatant is discarded and pancreatic enzyme is added to facilitate cell digestion. Once the cells acquire a rounded morphology, the reaction is terminated by adding culture medium. Following this, the cell mixture is aspirated using a pipette gun and transferred into a centrifuge tube for subsequent removal of the supernatant through centrifugation. Finally, third generation cells are selected for further experimental procedures.

### Cell uptake

Logarithmic growth phase cells were seeded at a density of 1×10^6^ cells/well in a 6-well culture plate, each well pre-equipped with a cell climbing slide. The cells were treated with CUR (40 μM) or CUR-NPs (CUR equivalent: 40 μM) and incubated at 37^o^C for 48 h. After removing the supernatant, the cells were washed three times with PBS and subsequently fixed in a formaldehyde solution (4%) at room temperature for 20 min. Following fixation, the cells were stained with 4,6-diamino-2-phenylindole (DAPI) for 10 min and their drug uptake was visualized using confocal laser scanning microscopy (CLSM; Olympus, Japan).

### Cell proliferation assay

Logarithmic growth phase cells were seeded at a density of 1×10^6^ cells/well in 96-well plates. Upon reaching 70% confluence, the cells were randomly allocated into control group, TGF-β1 group (10 ng/mL), TGF-β1 (10 ng/mL) + blank carrier group (concentration: 0–1000 μM), TGF-β1 (10 ng/mL) +CUR-NPs group (CUR equivalent: 0–50 μM). Following treatment, the cells in each group were further cultured at 37^o^C for an additional 48 h. Subsequently, CCK-8 reagent was added and incubated for another 2 h. The absorbance of each well was measured at a wavelength of 450 nm to determine the cell proliferation rate.

### Cell scratch assay

Logarithmic growth phase cells (1×10^6^ cells/well) were seeded in a 6-well plate and subjected to scratch treatment when cell confluence reached 70%. The cells were randomly allocated into control group, TGF-β1 group (10 ng/mL), and TGF-β1 (10 ng/mL)+CUR-NPs (CUR equivalent: 40 μM) group for administration. Incubation was carried out at 37^o^C for 48 h, followed by capturing microscopic images. Cell migration rate (%)=(scratch area in the drug treatment group/scratch area in the control group)×100.

### Transwell assay

Logarithmic growth phase cells (2×10^6^ cells/well) were inoculated into the upper chamber of a transwell and randomly divided into three groups: control group, TGF-β1 group (10 ng/mL), and TGF-β1 (10 ng/mL)+CUR-NPs group (CUR equivalent: 40 μM). After adding each drug to the lower chamber, incubation was carried out at 37^o^C for 48 h. The lower chamber was then removed, and the cells and culture medium in the upper chamber were wiped with a cotton swab. Subsequently, 4% paraformaldehyde was added to fix them at room temperature for 30 min. Staining was performed using 600 μL of 0.1% crystal violet solution at room temperature for 10 min, followed by cleaning with PBS and air drying. Five different fields of view were captured under a microscope for observation and photography, while the number of migrated cells in each group (stained purple) was calculated. ImageJ software was used to quantify cell migration through polycarbonate membranes.

### Detection of protein expression in mouse ASMCs

Logarithmic growth phase cells were seeded at a density of 2×10^6^ cells/dish in a cell culture dish. The experimental groups included the control group, TGF-β1 group (10 ng/mL), and TGF-β1 (10 ng/mL)+CUR-NPs (CUR equivalent: 40 μM) group. Following drug administration, the cells were harvested, and protein extraction was performed using a BCA kit to determine protein concentration. Subsequently, the proteins were denatured by incubating at 95^o^C for 5 min to obtain the protein samples. Take 40 μg of protein sample and perform SDS-PAGE under electrophoresis conditions of 100 V and 80 mA. Transfer the proteins from the gel to a PVDF membrane (0.45 μm) using transfer conditions of 25 V and 1.0 A. Subsequently, block with a solution containing 5% skim milk for 1 h, followed by incubation with primary antibodies including TGF-β1, total-STAT3, p-STAT3, CTGF, and β-actin (dilution ratio is 1:10000) at a temperature of 4^o^C overnight. Wash three times in total with TBST solution for 10 min each time. Then add horseradish peroxidase labeled goat anti-rabbit IgG secondary antibody (dilution ratio is 1:10000), incubate at room temperature for 1 h, wash three times in total with TBST solution for 10 min each time. After color development, use a gel imaging analysis system for imaging purposes and analyze the images using ImageJ version 1.8.0 image software tool to quantify the relative expression level of target proteins utilizing b-actin as an internal reference.

### Preparation and administration of asthma mouse models

The mice were randomly allocated into three groups: the control group, asthma model group, and asthma + CUR-NPs group. Mice in the asthma model group and asthma + CUR-NPs group received intraperitoneal injections of 20 μg ovalbumin (OVA) on days 0, 14, 28, and 42 of the experiment, followed by aerosolized stimulation on day 21. A nebulizer was used to administer a 1% OVA solution for inhalation lasting for 30 min every other day, three times a week (from days 21–46). Mice in the control group received intraperitoneal injections of an equal volume of saline and underwent aerosol inhalation. Mice in the asthma + CUR-NPs group were intravenously injected with 12.5 mg/kg, 25 mg/kg, and 50 mg/kg of equivalent CUR-NPs on a daily basis from initial sensitization until week 4 (21–49). CUR-NPs were injected via the tail vein 1 h prior to each stimulation throughout the entire stimulation period.

### Detection of TGF-β1 levels in serum of asthmatic mice

Extract blood from the eyeball and allow it to coagulate at room temperature for 1 h. Subsequently, centrifuge the collected blood for 10 min and collect the upper serum in a centrifuge tube for future use. Take 20 μL of the serum sample and mix it well with 480 μL of buffer solution, following the instructions provided by TGF-β1 ELISA kit.

### Detection of protein expression in lung tissue of asthmatic mice

After euthanizing the mice, the thoracic and abdominal cavities were opened, followed by excision of the right atrial appendage. Pre-cooled sterile physiological saline was then aspirated using a syringe and injected into the left ventricle for perfusion. Perfusion was terminated upon liver lightening to a pale yellow hue, after which lung tissue was dissected. Some samples were flash-frozen in liquid nitrogen and stored in an ultra-low temperature refrigerator, while others were fixed with 4% paraformaldehyde for subsequent experimental procedures. The tissue block was finely fragmented, followed by addition of lysate in accordance with the mass and volume of each sample. The protein sample was prepared and subjected to protein electrophoresis, PVDF conversion, non-specific blocking, and antibody incubation (including TGF-β1, total-STAT3, p-STAT3, CTGF as primary antibodies). Subsequently, chemiluminescence reagent kit was used for development and the optical density value of the target strip was analyzed using Gel Pro Analyzer software for gel image processing.

### Statistical analysis

The values are presented as mean ± standard deviation. Statistical analysis was performed using SPSS 19.0 software, and one-way analysis of variance (ANOVA) was employed to assess the statistical significance of the data. *p < 0.05 denotes significant differences.

## Results

### Basic parameters of CUR-NPs

**FIGURE 1:**
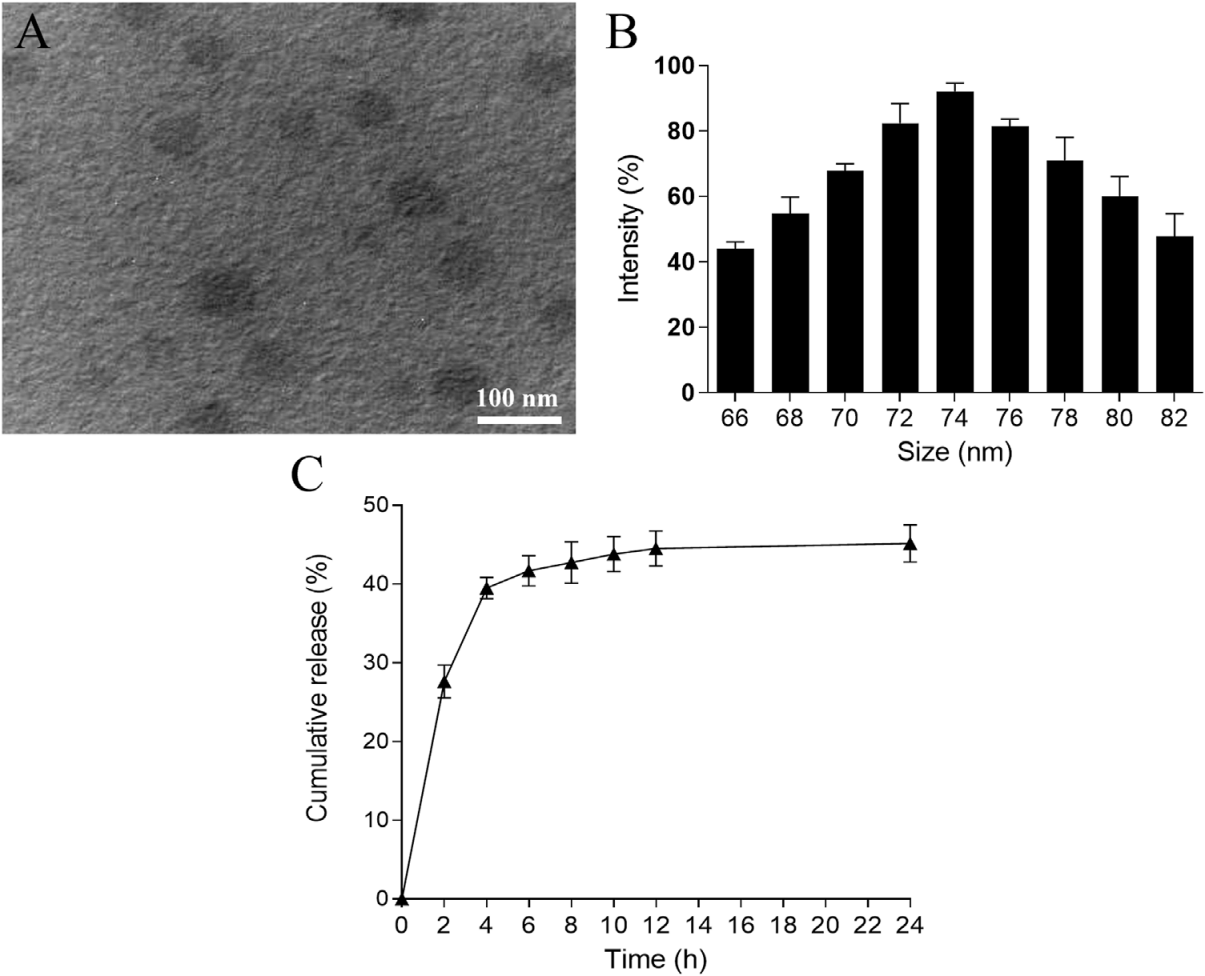
Characterization of CUR-NPs. TEM image **(A)** and size distribution **(B)** of CUR-NPs. **(C)** Cumulative amount of CUR released from CUR-NPs. TEM, transmission electron microscopy; CUR, curcumin; NPs, nanoparticles.

In this study, we aimed to synthesize CUR-NPs with a small size, high DL, and reasonable LE. Through orthogonal experiments, the optimization of nanoparticles preparation primarily focused on three factors: particle size, DL, and LE. The optimized conditions are summarized in the experimental section. Representative TEM images of the best-prepared CUR-NPs are shown in Figure 1A, demonstrating their sub-100 nm size and excellent uniformity. The size distribution of CUR-NPs is approximately Gaussian, as depicted in Figure 1B. Various parameters including average size, polydispersity index, potential, DL, and LE were calculated and are presented in Table 1. These results demonstrate that the CUR-NPs possess small particle size, nearly neutral surface charge properties, and high drug DL, indicating their promising potential for practical applications. The release profile of CUR-NPs is illustrated in Figure 1C. The curve demonstrates an initial rapid release of CUR from CUR-NPs within the first few hours, with approximately 40% of the cumulative amount released within 4 h. Subsequently, the release rate of CUR-NPs significantly decelerates and reaches a plateau phase after 12 h.

**TABLE 1:** Parameters for CUR-NPs.

Sample name	Size (nm)	Polydispersity index	Potential (mV)	DL (wt%)	LE (%)
CUR-NPs	74.05 ± 5.67	0.19	0.41 ± 0.013	8.81 ± 0.92	81.8 ± 4.42

CUR, curcumin; NPs, nanoparticles; DL, drug loading; LE, loading efficiency.

### Endocytosis of CUR-NPs

**FIGURE 2:**
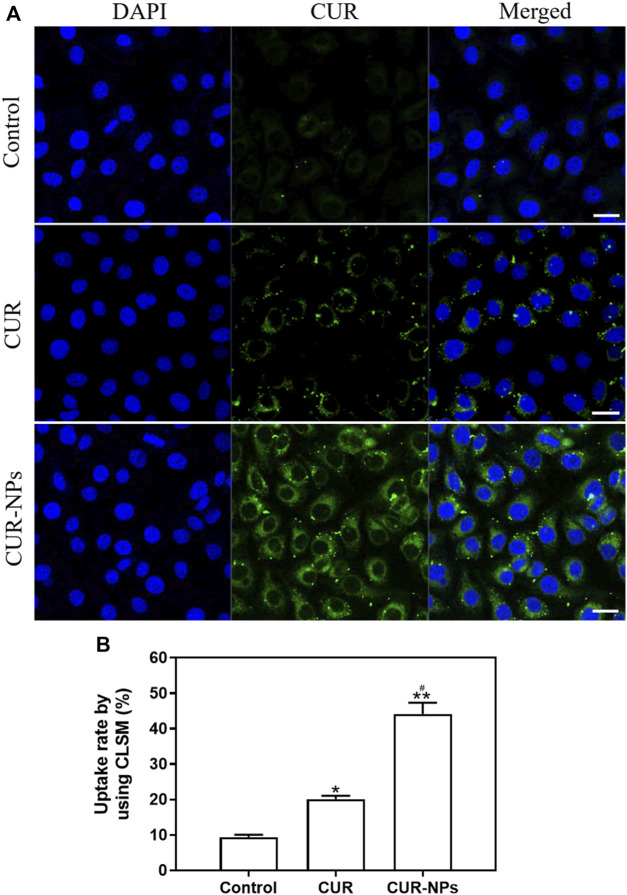
Cell uptake of CUR or CUR-NPs. **(A)** Cells were treated with CUR and CUR-NPs for 48 h, and drug uptake was measured using CLSM (CUR equivalent: 40 μM; scale bar: 50 μm). Green fluorescence: from CUR. Blue fluorescence: from DAPI. **(B)** Fluorescence intensity of cells treated with CUR and CUR-NPs for 48 h, respectively (CUR equivalent: 40 μM). Quantitative determination of fluorescence intensity. *p < 0.05, comparison of control group, **p < 0.01, comparison of control group, ^#^ *p* < 0.05, comparison with CUR group. CLSM, confocal laser scanning microscope; DAPI, 4,6-diamino-2-phenylindole; CUR, curcumin; NPs, nanoparticles.

To assess cellular uptake, cells were incubated with CUR (40 μM) or CUR-NPs (CUR equivalent: 40 μM) for 48 h and imaged using CLSM. As depicted in Figure 2A, the fluorescence intensity of cells treated with CUR-NPs was significantly higher than that of those treated with free CUR. The obtained images provide compelling evidence for the remarkable enhancement of intracellular CUR accumulation achieved by the currently developed CUR-NPs. As depicted in Figure 2B, statistical analysis further confirms a significantly higher intracellular uptake rate of CUR-NPs compared to that of free CUR, thereby fully substantiating the inherent advantages offered by nanoscale carriers. The uptake of CUR-NPs represents the initial step in intracellular action, making it a pivotal discovery in this study for elucidating their mechanism of action.

### Impact of blank carriers or CUR-NPs on TGF-β1-induced cellular proliferation

**FIGURE 3:**
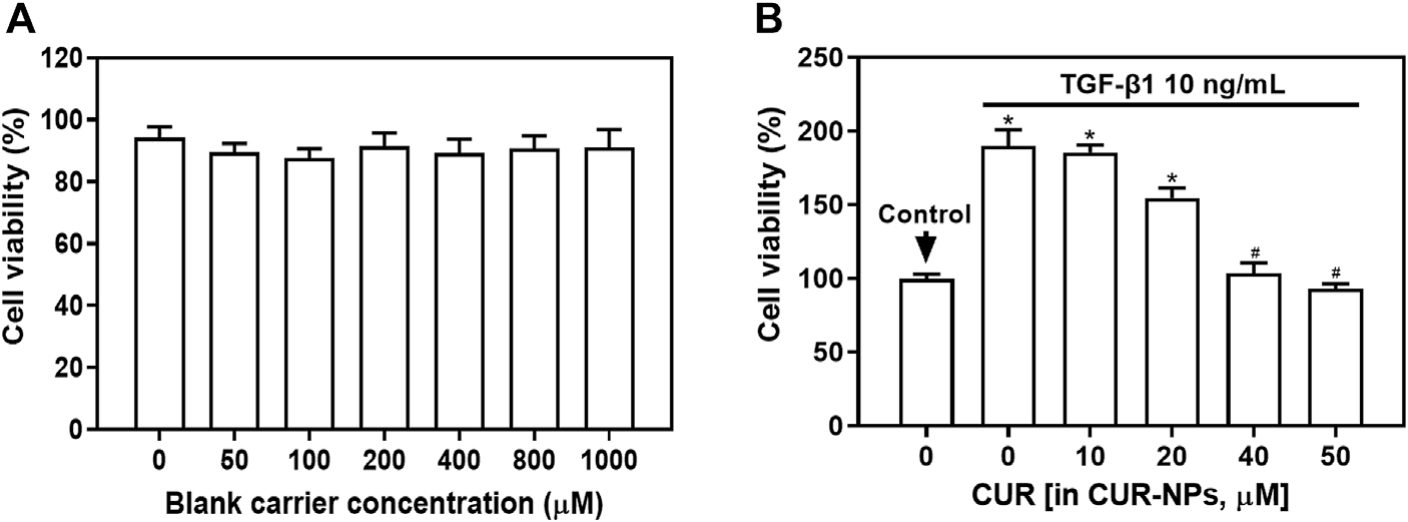
Effect of blank carriers or CUR-NPs on TGF-β1-induced cell proliferation. **(A)** The effect of blank carriers with different concentrations on TGF-β1-induced cell proliferation. **(B)** The effect of different concentrations of CUR-NPs on TGF-β1-induced cell proliferation. *p < 0.05, comparison of control group, ^#^ *p* < 0.05, comparison with CUR group. CUR, curcumin; NPs, nanoparticles.

In order to elucidate the impact of blank carriers in the nanoparticle system on therapeutic efficacy, we performed cell proliferation detection using the CCK-8 assay. As depicted in Figure 3A, TGF-β1-induced cell proliferation exhibited a significant increase. Interestingly, even with escalating concentrations of blank carriers (0–1000 μM), no discernible decline in TGF-β1-mediated cell proliferation was observed. Notably, all treatment groups displayed cell proliferation rates exceeding 90%, suggesting that the presence of blank carriers had negligible influence on cellular growth.

We employed the CCK-8 assay to assess the impact of varying concentrations of CUR-NPs on TGF-β1-induced cellular proliferation. As depicted in Figure 3B, the TGF-β1 group exhibited a significant upsurge in cell proliferation (*p* < 0.05). Notably, an inverse correlation was observed between the concentration of CUR-NPs and TGF-β1-induced cell proliferation. When the concentration of CUR equivalent was 40 μM, the proliferation rate of TGF-β1+CUR-NPs group cells exhibited a significant decrease (*p* < 0.05), but no statistically significant difference was observed compared to the group with a CUR equivalent concentration of 50 μM (*p* > 0.05). These findings suggest that CUR-NPs (with CUR equivalents of 40 μM and 50 μM) possess inhibitory effects on cell proliferation without demonstrating enhanced inhibition at higher concentrations. Therefore, we opted for a CUR-NPs administration concentration of 40 μM as the CUR equivalent and selected a drug treatment duration of 48 h for cell scratch assay, transwell assay, and Western blot assay.

### Impact of CUR-NPs on TGF-β1-induced cellular migration

**FIGURE 4:**
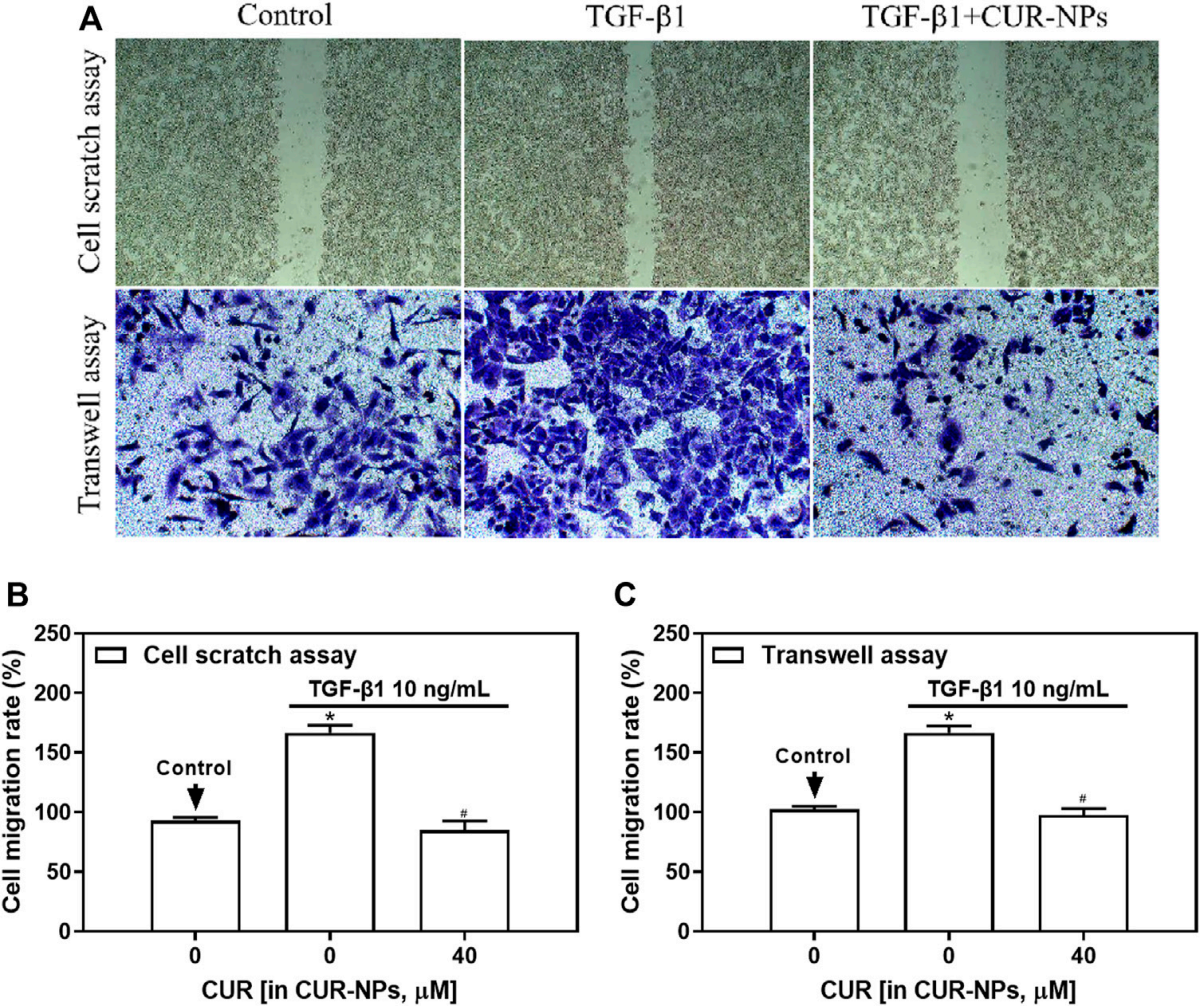
Effect of CUR-NPs on TGF-β1-induced cell migration. **(A)** Picture results of cell scratch assay and transwell assay. **(B)** Calculate cell migration rate through statistical analysis of cell scratch assay. **(C)** Calculate cell migration rate through statistical analysis of transwell assay. *p < 0.05, comparison of control group; ^#^ *p* < 0.05, comparison with TGF-β1 group. CUR, curcumin; NPs, nanoparticles.

We initially assessed the impact of CUR-NPs on TGF-β1-induced cellular migration using a cell scratch assay, as depicted in Figure 4A and Figure 4B. The TGF-β1 group exhibited a substantial augmentation in cellular migration, whereas the TGF-β1+CUR-NPs (CUR equivalent: 40 μM) group demonstrated a significant reduction in cellular migratory capacity, thereby indicating the pronounced inhibitory effect of CUR-NPs on cellular migration. The same results were also confirmed in the transwell assay, as depicted in Figure 4A and Figure 4C. Specifically, the TGF-β1 group exhibited a significant augmentation in cell migration (*p* < 0.05), whereas the TGF-β1+CUR-NPs (CUR equivalent: 40 μM) group demonstrated a substantial attenuation in cellular migratory capacity towards the lower chamber (*p* < 0.05). This corroborates our previous findings and further substantiates that CUR-NPs exert a pronounced inhibitory effect on cell migration.

### Impact of CUR-NPs on the expression of proteins associated with the intracellular TGF-β1 signaling pathway

**FIGURE 5:**
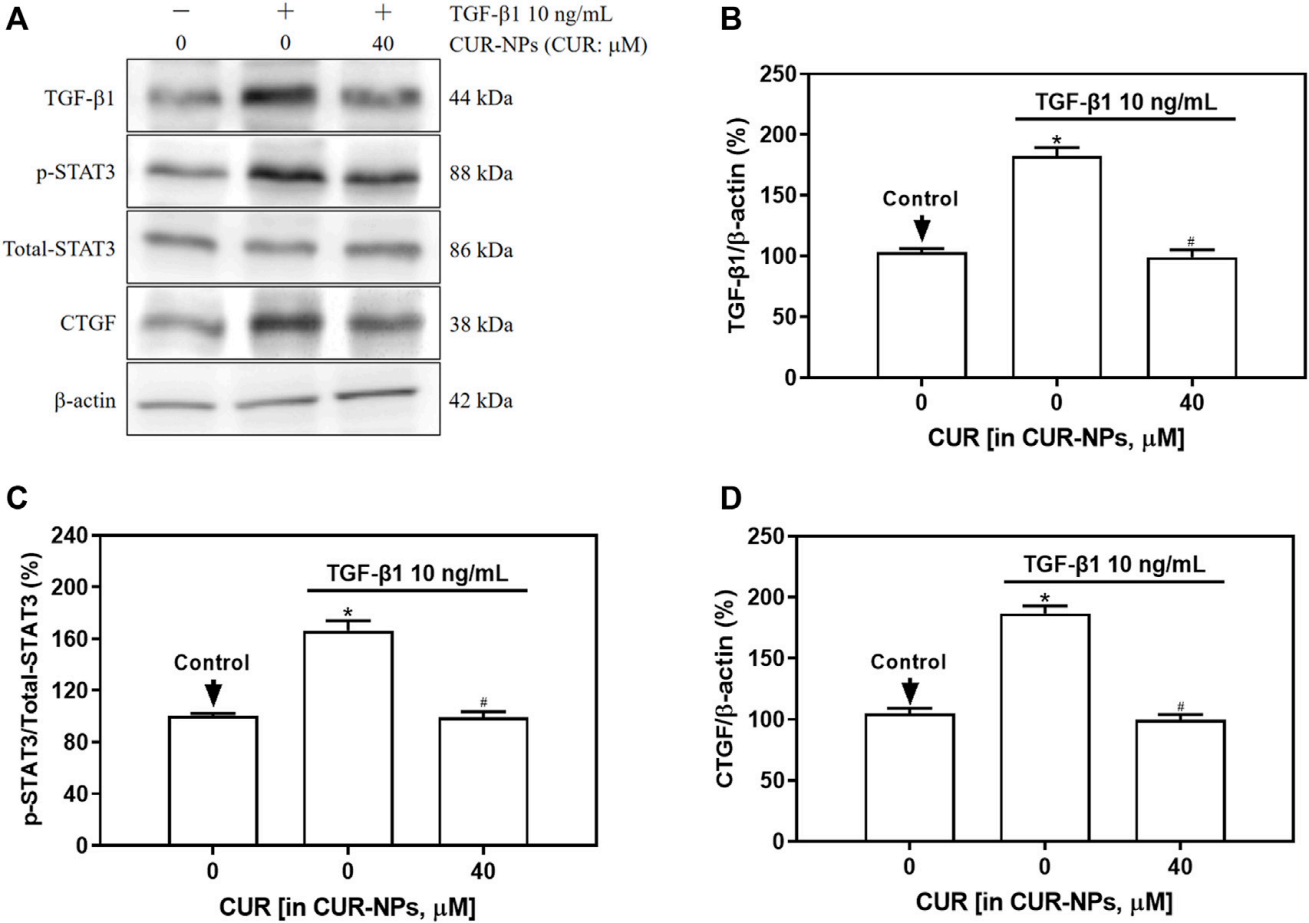
Effect of CUR-NPs on TGF-β1 signaling pathway related protein expression. **(A)** Representative bands of TGF-β1, total-STAT3, p-STAT3, CTGF, β-actin. **(B)** Quantitative determination of TGF-β1/β-actin ratio. **(C)** Quantitative determination of p-STAT3/total-STAT3 ratio. **(D)** Quantitative determination of CTGF/β-actin ratio.*p < 0.05, comparison of control group; ^#^ *p* < 0.05, comparison with TGF-β1 group. CUR, curcumin; NPs, nanoparticles.

In this study, Western blotting was employed to assess the impact of CUR-NPs on intracellular TGF-β1, total-STAT3, p-STAT3, and CTGF protein expression induced by TGF-β1. As depicted in Figure 5, the levels of TGF-β1, p-STAT3, and CTGF proteins were significantly upregulated in the TGF-β1 group (*p* < 0.05). In the TGF-β1+CUR-NPs (CUR equivalent: 40 μM) group, a significant reduction in the expression of TGF-β1, p-STAT3, and CTGF was observed (*p* < 0.05). There were no notable differences in total-STAT3 expression among the control group, TGF-β1 group, and TGF-β1+CUR-NPs (CUR equivalent: 40 μM) group (*p* > 0.05).

### Impact of CUR-NPs on serum levels of TGF-β1 in asthmatic mice

**FIGURE 6:**
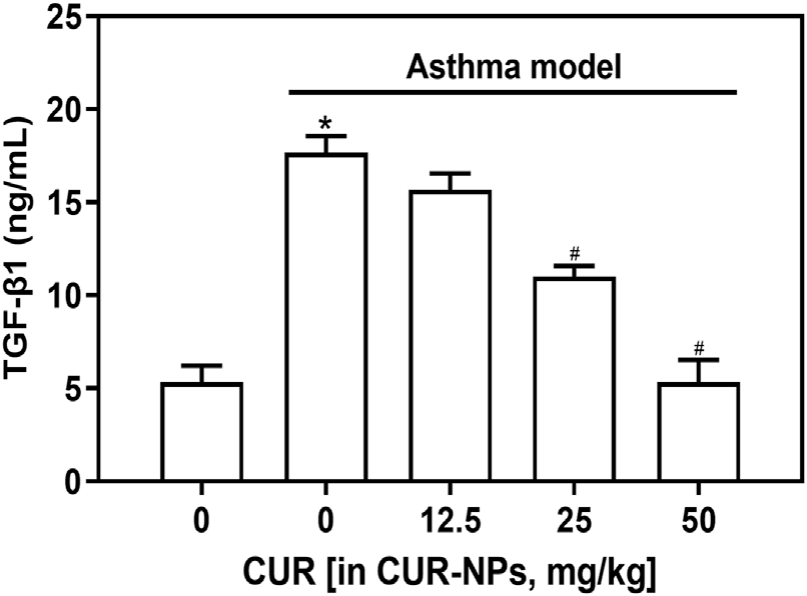
Effect of CUR-NPs on the levels of TGF-β1 in the serum of asthma mice. *p < 0.05, comparison of control group; ^#^ *p* < 0.05, comparison with asthma model group. CUR, curcumin; NPs, nanoparticles.

ELISA was employed to quantify the levels of TGF-β1 in the serum samples from each group of mice. As depicted in Figure 6, a significant increase in TGF-β1 levels was observed in the asthma model group compared to the control group (*p* < 0.05). Furthermore, an incremental decrease in serum TGF-β1 content was observed with increasing doses of CUR-NPs in the asthma + CUR-NPs group. The serum TGF-β1 levels in the asthma + CUR-NPs (CUR equivalent: 25 mg/kg) and asthma + CUR-NPs (CUR equivalent: 50 mg/kg) groups of mice exhibited significant differences compared to the asthma model group (*p* < 0.05). For the subsequent *in vivo* experiment, we will utilize a dosage of 25 mg/kg as the treatment group’s CUR equivalent.

### Impact of CUR-NPs on the expression of proteins related to the TGF-β1 signaling pathway in lung tissue of asthmatic mice

**FIGURE 7:**
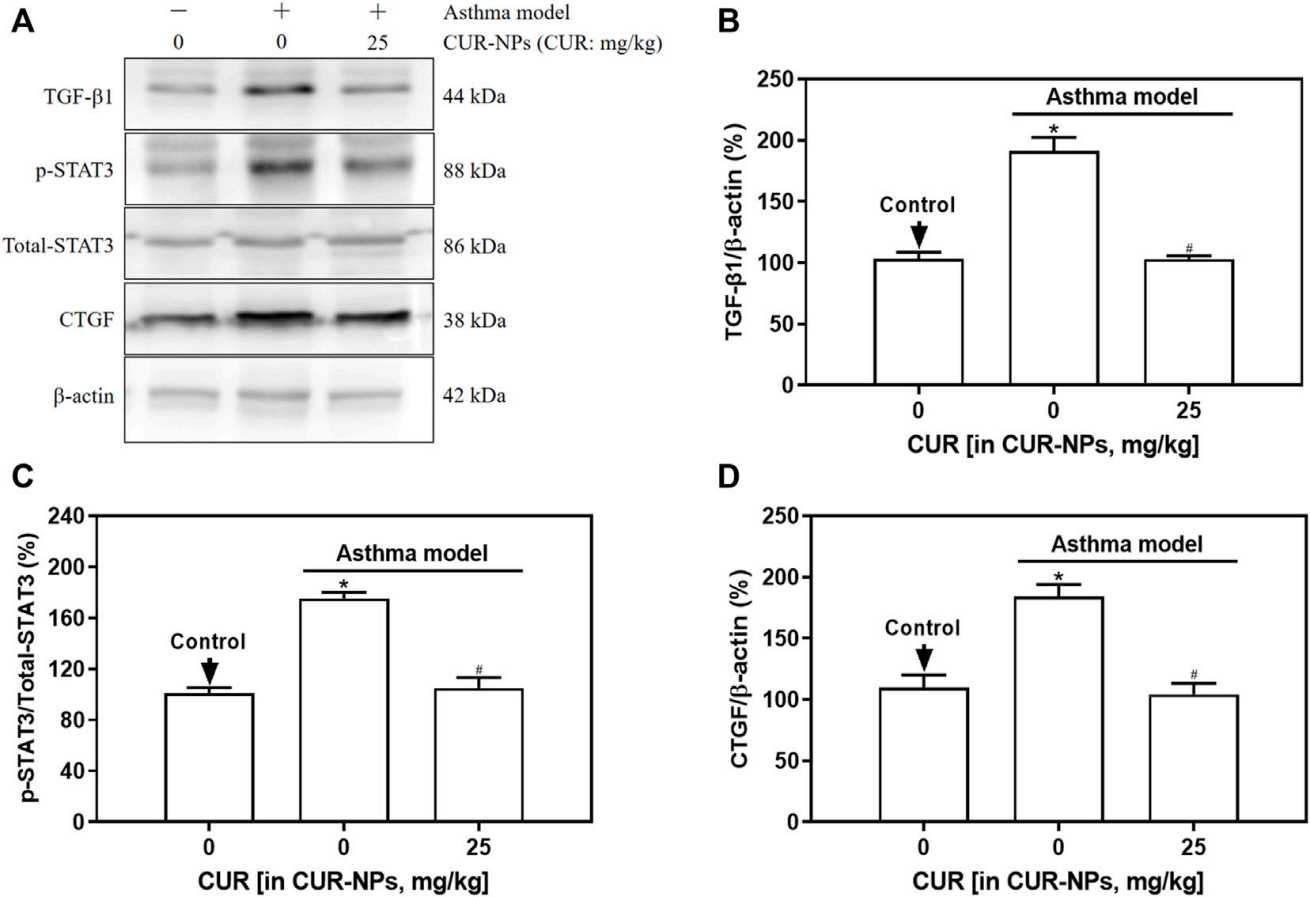
Effect of CUR-NPs on TGF-β1 signaling pathway related protein expression in lung tissue of asthmatic mice. **(A)** Representative bands of TGF-β1, total-STAT3, p-STAT3, CTGF, β-actin. **(B)** Quantitative determination of TGF-β1/β-actin ratio. **(C)** Quantitative determination of p-STAT3/total-STAT3 ratio. **(D)** Quantitative determination of CTGF/β-actin ratio. *p < 0.05, comparison of control group; ^#^ *p* < 0.05, comparison with asthma model group. CUR, curcumin; NPs, nanoparticles.

The protein expression levels of TGF-β1, p-STAT3, and CTGF in the lung tissue of asthma mice were significantly elevated compared to those in the control group (*p* < 0.05), as demonstrated by Western blot analysis (Figure 7). The protein expression levels of TGF-β1, p-STAT3, and CTGF in the lung tissue of mice in the asthma + CUR-NPs group (CUR equivalent: 25 mg/kg) were significantly lower compared to those in the asthma model group (*p* < 0.05). However, there was no significant difference when compared to the control group (*p* > 0.05). There was no statistically significant difference observed in the expression level of STAT3 protein in the lung tissue between the asthma model group and the asthma + CUR-NPs (CUR equivalent: 25 mg/kg) group, when compared to the control group (*p* > 0.05).

## Discussion

Bronchial asthma (asthma) is a prevalent chronic non-infectious respiratory disease characterized by chronic airway inflammation involving various cellular components (Hough et al., 2020). It presents clinically as recurrent wheezing, dyspnea, with or without chest tightness or cough, accompanied by airway hyperresponsiveness and variable airflow limitation (Fehrenbach et al., 2017). With disease progression, it can lead to airway remodeling, which refers to structural changes in the airways (Dhanjal et al., 2022). Airway smooth muscle consists of smooth muscle cells and intercellular connective tissue, constituting a normal component of both large and small airways (Ramos-Ramírez and Tliba, 2022). It plays a crucial role in regulating airway reactivity and maintaining bronchial tension as well as pulmonary ventilation (Fehrenbach et al., 2017). Studies have demonstrated that airway smooth muscle cells play a pivotal role in regulating airway function through their ability to contract and relax, as well as contribute to airway remodeling via cellular proliferation, thereby leading to the development of airway stenosis in individuals with asthm (Yang et al., 2023). Furthermore, in response to external stimuli, the activation of G protein-coupled receptors, receptor tyrosine kinases, and integrins initiates signal transduction cascades within downstream airway smooth muscle cells (Deng et al., 2008). This cascade leads to polarization, protrusion, adhesion, traction, and contraction of airway smooth muscle cells for facilitating their migration and subsequently inducing airway remodeling. It is evident that the proliferation and migration of airway smooth muscle cells play a pivotal role in promoting the augmentation of airway smooth muscle mass in individuals with asthma. The augmented secretion of diverse inflammatory factors, cytokines, and growth factors in asthmatic patients significantly contributes to the stimulation of airway smooth muscle cell proliferation and migration (Howell and Mc Anulty, 2006; Yang et al., 2012). In recent years, TGF-β has garnered considerable attention as a growth factor capable of fostering airway smooth muscle cell proliferation and migration (Chen et al., 2016; Chen Q et al., 2018).

TGF-β is a cytokine secreted by immune and epithelial cells, with three subtypes identified, among which TGF-β1 is the most widely expressed subtype (Poniatowski et al., 2015; Ojiaku et al., 2017). TGF-β1 is closely implicated in the pathogenesis and progression of asthma, exerting both anti-inflammatory and pro-inflammatory effects on the inflammatory response. Additionally, TGF-β1 serves as a pivotal mediator in tissue remodeling processes such as fibrosis (Devan et al., 2022). The expression of TGF-β1 in the lung tissue of mice or rats with asthma airway remodeling has been found to be significantly increased (Liu et al., 2015). Additionally, researchers have observed that TGF-β1 can stimulate the proliferation and migration of airway smooth muscle cells (Chen et al., 2016; Chen Q et al., 2018), leading to bronchial subepithelial thickening and collagen deposition, ultimately resulting in bronchial stenosis in patients with asthma. TGF-β1 plays a pivotal role in epithelial mesenchymal transdifferentiation primarily through the canonical Smad pathway and other signaling cascades (Zhang et al., 2012). In the canonical pathway, TGF-β1 dissociates from the latency-associated protein complex upon receiving an activating signal, subsequently binding to type I TGF-β1 receptor to initiate the process of epithelial mesenchymal transdifferentiation (Zhang et al., 2012). The activated TGF-β1 receptor can initiate the activation of downstream signaling pathways, such as the STAT3 pathway (Xu et al., 2014). Previous studies have demonstrated the involvement of STAT3 signaling pathway activation in airway remodeling during asthma (Wang et al., 2023). The STAT3 signaling pathway is implicated in tissue repair and the regulation of collagen deposition, with sustained activation of STAT3 resulting in excessive tissue repair (Kasembeli et al., 2018). Previous studies have demonstrated that activation of the STAT3 signaling pathway can enhance cellular proliferation and migration capabilities (Yao et al., 2015). Additionally, TGF-β1 can induce cell differentiation and collagen deposition through the activation of the STAT3 signaling pathway (Xu et al., 2014). Recently conducted studies have demonstrated that CTGF plays a pivotal role in facilitating the extracellular matrix formation during airway remodeling, while also exhibiting the ability to stimulate smooth muscle cell proliferation and migration (Gao et al., 2017). Moreover, it has been observed that TGF-β1 can upregulate the expression of CTGF protein in asthmatic smooth muscle cells (Wang et al., 2018). Furthermore, several investigations have substantiated that CTGF serves as a novel fibrogenic protein induced by TGF-β, acting as a downstream mediator of this signaling pathway. Additionally, studies have demonstrated that TGF-β1 can activate the STAT3 signaling pathway in hepatocytes and induce the release of CTGF (Makino et al., 2023). Consequently, the activation of the STAT3 signaling pathway along with TGF-β1 and CTGF is implicated in cellular proliferation and migration, thereby influencing airway remodeling. Hence, TGF-β1 was employed to stimulate ASMCs’ proliferation and migration in this investigation. Our experimental findings demonstrate that the TGF-β1 group exhibited significantly higher rates of cell proliferation and migration compared to the control group, indicating successful induction of ASMCs’ proliferation and migration by TGF-β1. Additionally, the protein expression levels of TGF-β1, p-STAT3, and CTGF in ASMCs were found to be significantly elevated in the TGF-β1 group compared to the control group. However, there was no observed change in the expression level of total-STAT3. The successful activation of the TGF-β1/p-STAT3/CTGF signaling pathway in ASMCs is indicated by these findings. In animal experiments, significantly elevated levels of TGF-β1 in the serum of asthmatic mice and increased protein expression levels of TGF-β1, p-STAT3, and CTGF in lung tissues were observed compared to the control group. The experimental results have confirmed the elevation of TGF-β1 levels and the activation of downstream p-STAT3 and CTGF signaling pathway proteins in asthmatic mice.

CUR, a plant polyphenol derived from the roots of Curcuma plant, serves as the principal bioactive compound responsible for its pharmacological effects (Maiti and Dunbar, 2018). Apart from its traditional properties, CUR exhibits notable anti-inflammatory, antioxidant, and anti-fibrotic activities (He et al., 2015). Therefore, CUR has emerged as a prominent area of research in the field of prevention and treatment for various inflammatory diseases, particularly respiratory disorders (Hassani et al., 2015). CUR effectively diminishes the proportion of inflammatory cells present in bronchoalveolar lavage fluid of asthmatic mice, attenuates eosinophilic infiltration, ameliorates airway inflammation in murine models, and mitigates excessive mucus production (Islam et al., 2022). In addition to its anti-inflammatory effects, CUR exerts regulatory effects on cytokines, thereby impeding cell proliferation and intercellular substance synthesis. Moreover, CUR suppresses epithelial hyperplasia and smooth muscle layer thickening while reducing collagen fiber deposition surrounding the airway and downregulating TGF-β1 expression. Gaedeke et al. (Gaedeke et al., 2004) demonstrated that CUR exerts its anti-fibrotic effect by inhibiting the expression of TGF-β1 and its receptor signaling pathway in renal fibroblasts. Furthermore, in bleomycin-induced pulmonary fibrosis experiments, CUR was found to reduce the expression of TGF-β1 and effectively inhibit the progression of pulmonary fibrosis (Xu et al., 2007). Revised sentence: Hu Y et al. (Hu et al., 2010) demonstrated that CUR effectively inhibits TGF-β1-induced proliferation of HK-2 cells. However, the clinical application and mechanism research of CUR are currently limited due to its poor water solubility, photodegradation, chemical instability, and low bioavailability. Therefore, it is imperative to develop appropriate drug delivery systems to address these challenges. In this study, the encapsulation of CUR in nanoparticles was employed to safeguard it against degradation and enhance its therapeutic efficacy. Furthermore, our uptake results substantiated that cellular drug accumulation was significantly augmented upon treatment with CUR-NPs compared to small molecule CUR, thereby affirming the superiority of nanoparticles. Meanwhile, we confirmed that the impact of the empty vector on cell proliferation was negligible, thereby establishing the insignificance of the vector itself. To assess the influence of CUR-NPs on ASMCs’ proliferation and migration, TGF-β1 was employed to induce these cellular processes. Importantly, CUR-NPs exhibited a significant inhibitory effect on TGF-β1-induced ASMCs’ proliferation and migration. We also assessed the protein expression levels of TGF-β1, total-STAT3, p-STAT3, and CTGF in ASMCs from each group and observed that CUR-NPs effectively attenuated the activation of the TGF-β1/p-STAT3/CTGF signaling pathway in ASMCs. *In vivo* study, we identified an upregulation of the TGF-β1/p-STAT3/CTGF signaling pathway in asthmatic mice; however, treatment with CUR-NPs significantly reduced the protein expression levels of TGF-β1, p-STAT3, and CTGF in lung tissue without affecting total-STAT3 expression level. These findings indicate that CUR-NPs possess inhibitory effects on the activation of the TGF-β1/p-STAT3/CTGF signaling pathway within asthmatic mouse lung tissue.

## Conclusions

**FIGURE 8:**
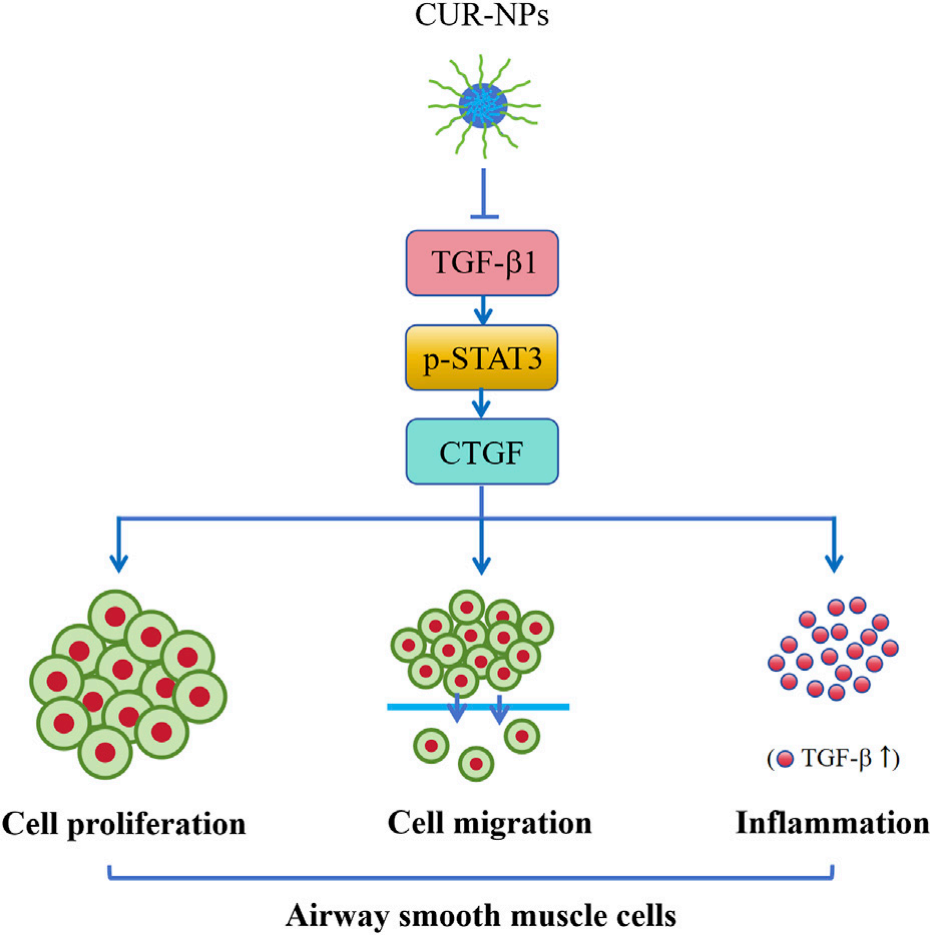
Mechanism of CUR-NPs. Stimulation of ASMCs by TGF-β1 enhances cell proliferation and migration, and activates the TGF-β1/p-STAT3/CTGF signaling pathway. Similarly, there is a significant increase in serum TGF-β1 level and expression levels of TGF-β1, p-STAT3, and CTGF proteins in lung tissue of asthmatic mice. However, CUR-NPs effectively mitigate this change through inhibiting the expression of TGF-β1 protein, thereby suppressing the downstream expression of p-STAT3 and CTGF proteins related to TGF-β1 signaling pathway, ultimately exerting an inhibitory effect on the proliferation, migration, and TGF-β1-related inflammatory infiltration of ASMCs. CUR, curcumin; NPs, nanoparticles; ASMCs, mouse airway smooth muscle cells.

In summary, CUR-NPs exhibit significant inhibitory effects on the proliferation and migration of airway smooth muscle cells, thereby effectively reversing airway remodeling in asthma. The underlying mechanism of action involves the inhibition of TGF-β1/p-STAT3/CTGF signaling pathway (Figure 8). Consequently, CUR-NPs hold great promise as a potential therapeutic agent for the treatment of asthma. However, it is important to acknowledge that this study did not include any pharmacokinetic or *in vitro* and *in vivo* toxicity studies on CUR-NPs. Therefore, further experiments are necessary to reinforce these conclusions. TGF-β1 is considered to be closely associated with the inflammatory response mechanism of asthma. In this study, while TGF-β1 was utilized as an assessment indicator for chronic airway inflammation, the elevated levels of TGF-β1 in the blood of asthmatic mice and the inhibitory effect of CUR-NPs on it were also observed. This finding demonstrates the inhibitory impact of CUR-NPs on TGF-β1-mediated airway inflammation. However, it does not represent the complete mechanism underlying the inhibitory effect of CUR-NPs on airway inflammation; therefore, further studies are needed to evaluate additional indicators in order to elucidate a more comprehensive and detailed mechanism.

## Data Availability

The original contributions presented in the study are included in the article/Supplementary Material, further inquiries can be directed to the corresponding author.
