# Supplementary material for: Effect of curcumin nanoparticles on proliferation and migration of mouse airway smooth muscle cells and airway inflammatory infiltration
Source: Front Pharmacol. 2024 Apr 19;15:1344333. doi: 10.3389/fphar.2024.1344333 (PMC11066239; doi:10.3389/fphar.2024.1344333)
Supplement: Supplementary file 1 [file Presentation1.PPTX]

## Slide 1
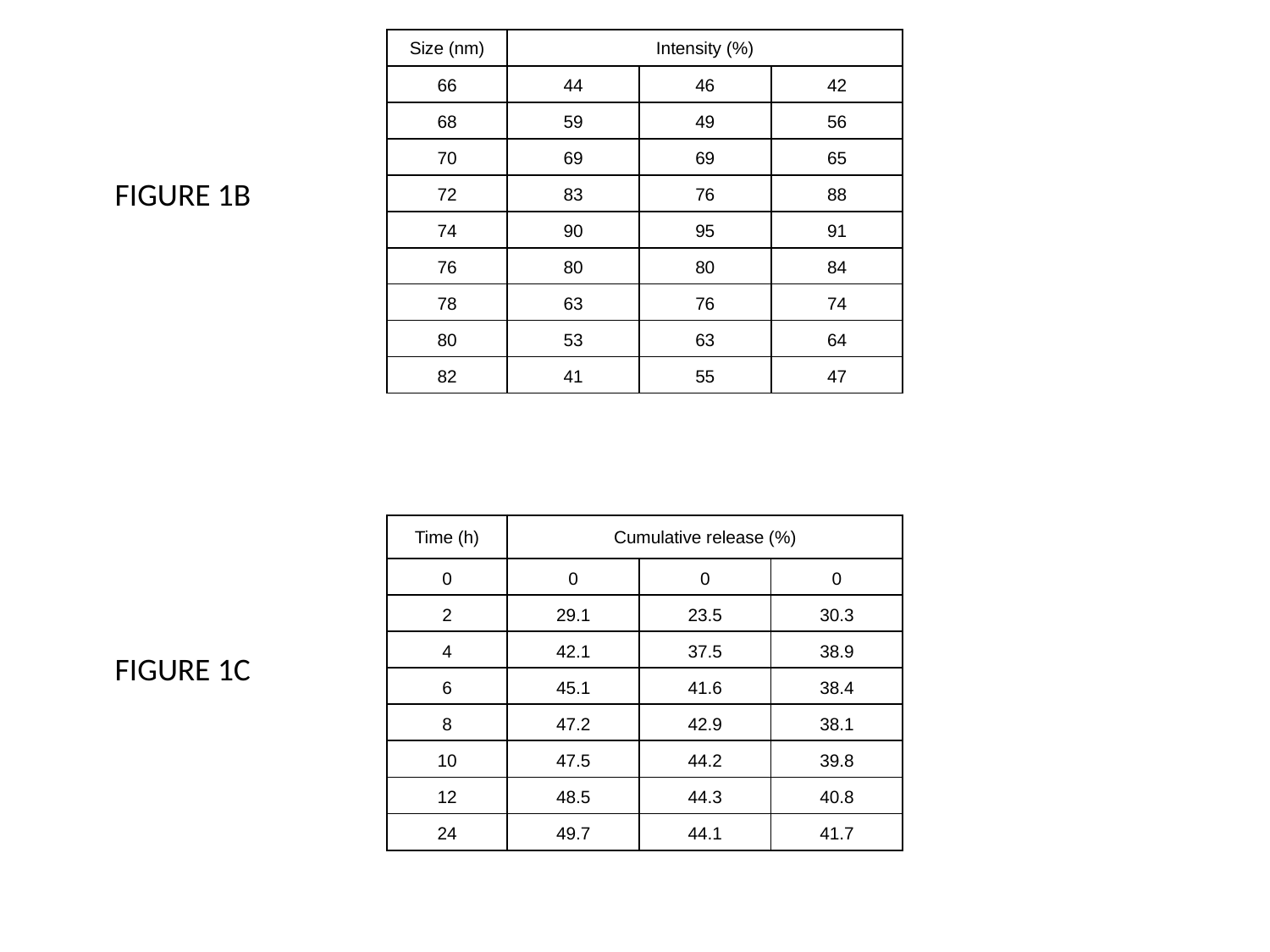

| Size (nm) | Intensity (%) | | |
| --- | --- | --- | --- |
| 66 | 44 | 46 | 42 |
| 68 | 59 | 49 | 56 |
| 70 | 69 | 69 | 65 |
| 72 | 83 | 76 | 88 |
| 74 | 90 | 95 | 91 |
| 76 | 80 | 80 | 84 |
| 78 | 63 | 76 | 74 |
| 80 | 53 | 63 | 64 |
| 82 | 41 | 55 | 47 |
FIGURE 1B
| Time (h) | Cumulative release (%) | | |
| --- | --- | --- | --- |
| 0 | 0 | 0 | 0 |
| 2 | 29.1 | 23.5 | 30.3 |
| 4 | 42.1 | 37.5 | 38.9 |
| 6 | 45.1 | 41.6 | 38.4 |
| 8 | 47.2 | 42.9 | 38.1 |
| 10 | 47.5 | 44.2 | 39.8 |
| 12 | 48.5 | 44.3 | 40.8 |
| 24 | 49.7 | 44.1 | 41.7 |
FIGURE 1C

## Slide 2
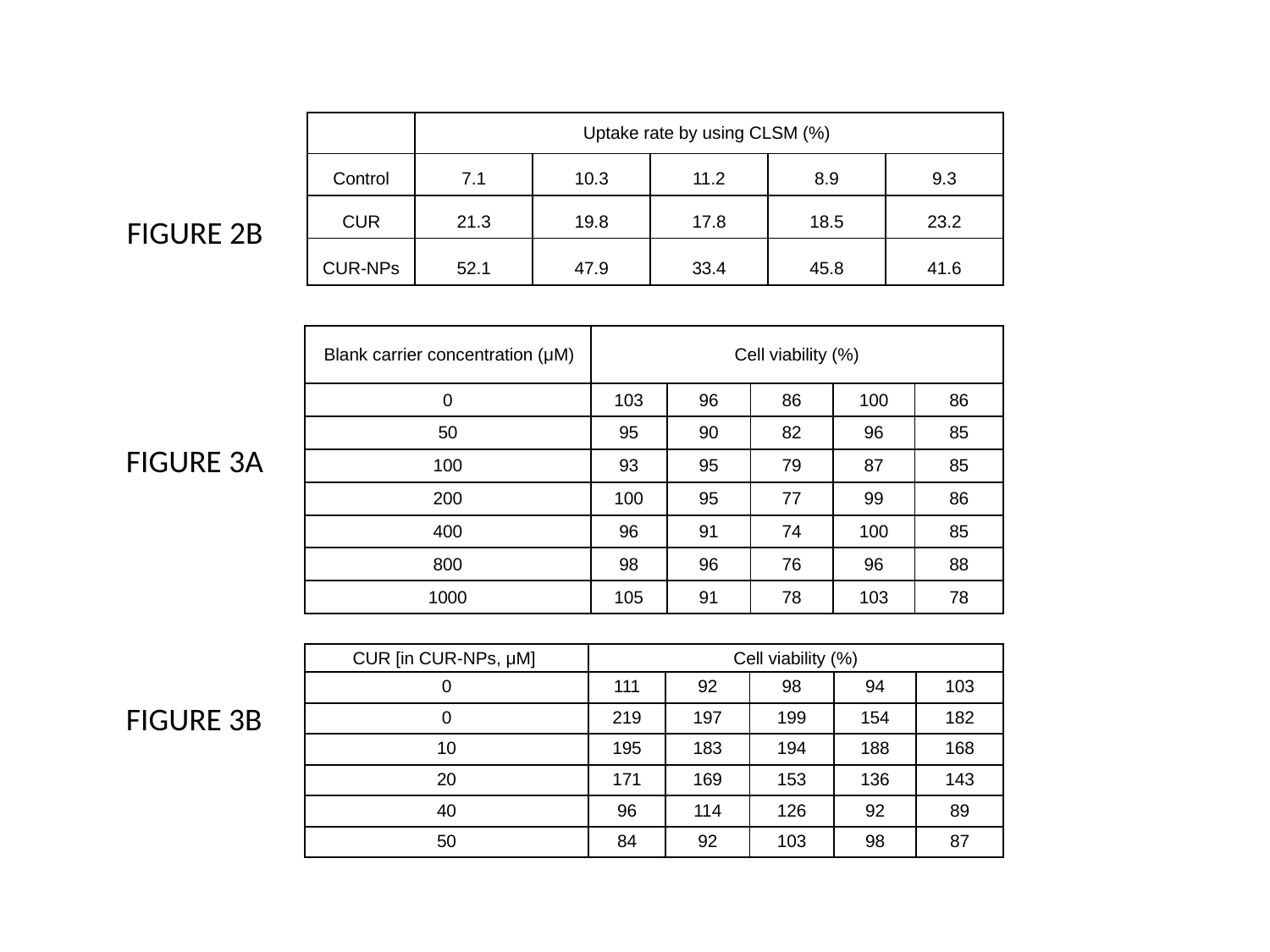

| | Uptake rate by using CLSM (%) | | | | |
| --- | --- | --- | --- | --- | --- |
| Control | 7.1 | 10.3 | 11.2 | 8.9 | 9.3 |
| CUR | 21.3 | 19.8 | 17.8 | 18.5 | 23.2 |
| CUR-NPs | 52.1 | 47.9 | 33.4 | 45.8 | 41.6 |
FIGURE 2B
| Blank carrier concentration (μM) | Cell viability (%) | | | | |
| --- | --- | --- | --- | --- | --- |
| 0 | 103 | 96 | 86 | 100 | 86 |
| 50 | 95 | 90 | 82 | 96 | 85 |
| 100 | 93 | 95 | 79 | 87 | 85 |
| 200 | 100 | 95 | 77 | 99 | 86 |
| 400 | 96 | 91 | 74 | 100 | 85 |
| 800 | 98 | 96 | 76 | 96 | 88 |
| 1000 | 105 | 91 | 78 | 103 | 78 |
FIGURE 3A
| CUR [in CUR-NPs, μM] | Cell viability (%) | | | | |
| --- | --- | --- | --- | --- | --- |
| 0 | 111 | 92 | 98 | 94 | 103 |
| 0 | 219 | 197 | 199 | 154 | 182 |
| 10 | 195 | 183 | 194 | 188 | 168 |
| 20 | 171 | 169 | 153 | 136 | 143 |
| 40 | 96 | 114 | 126 | 92 | 89 |
| 50 | 84 | 92 | 103 | 98 | 87 |
FIGURE 3B

## Slide 3
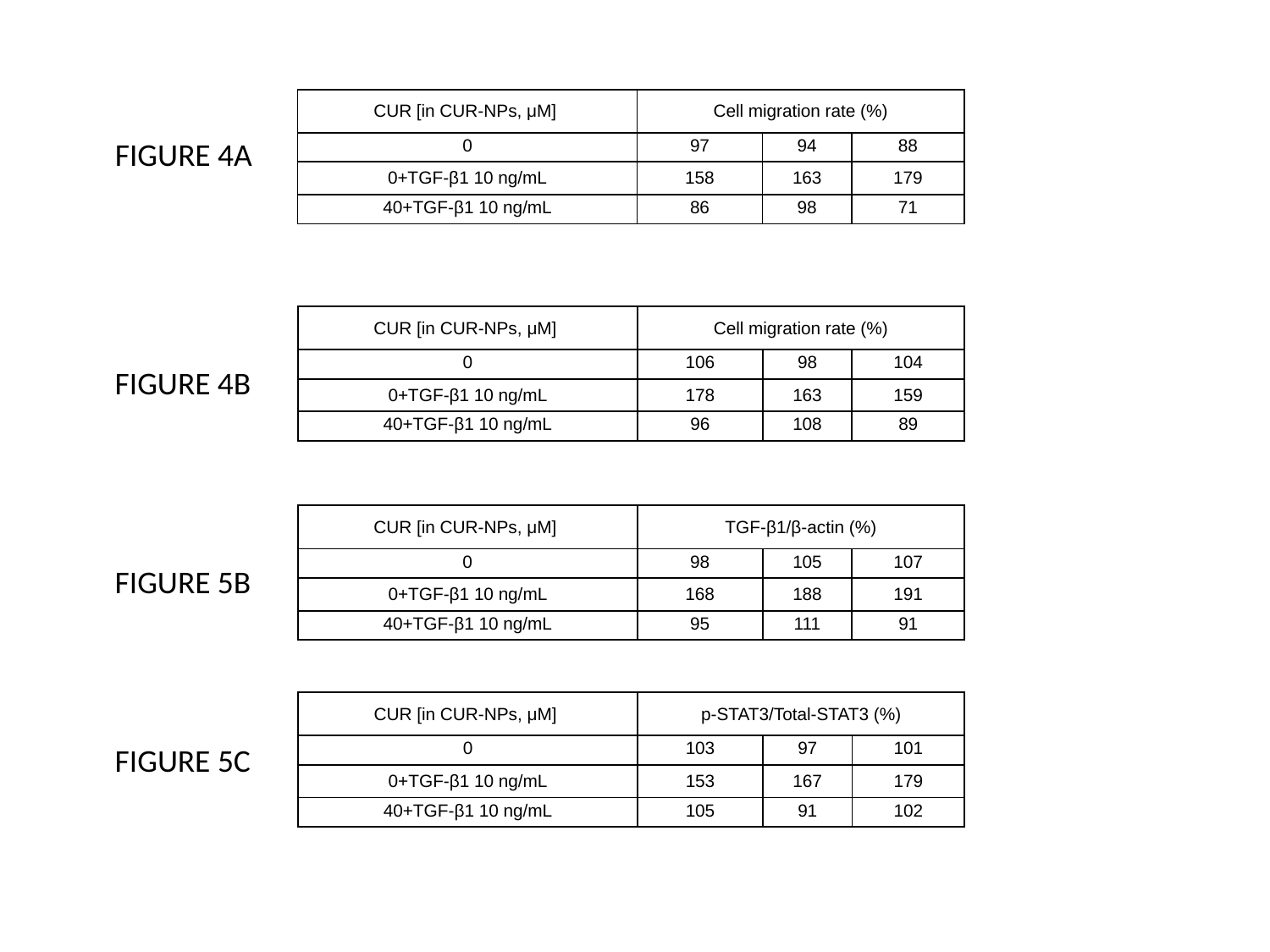

| CUR [in CUR-NPs, μM] | Cell migration rate (%) | | |
| --- | --- | --- | --- |
| 0 | 97 | 94 | 88 |
| 0+TGF-β1 10 ng/mL | 158 | 163 | 179 |
| 40+TGF-β1 10 ng/mL | 86 | 98 | 71 |
FIGURE 4A
| CUR [in CUR-NPs, μM] | Cell migration rate (%) | | |
| --- | --- | --- | --- |
| 0 | 106 | 98 | 104 |
| 0+TGF-β1 10 ng/mL | 178 | 163 | 159 |
| 40+TGF-β1 10 ng/mL | 96 | 108 | 89 |
FIGURE 4B
| CUR [in CUR-NPs, μM] | TGF-β1/β-actin (%) | | |
| --- | --- | --- | --- |
| 0 | 98 | 105 | 107 |
| 0+TGF-β1 10 ng/mL | 168 | 188 | 191 |
| 40+TGF-β1 10 ng/mL | 95 | 111 | 91 |
FIGURE 5B
| CUR [in CUR-NPs, μM] | p-STAT3/Total-STAT3 (%) | | |
| --- | --- | --- | --- |
| 0 | 103 | 97 | 101 |
| 0+TGF-β1 10 ng/mL | 153 | 167 | 179 |
| 40+TGF-β1 10 ng/mL | 105 | 91 | 102 |
FIGURE 5C

## Slide 4
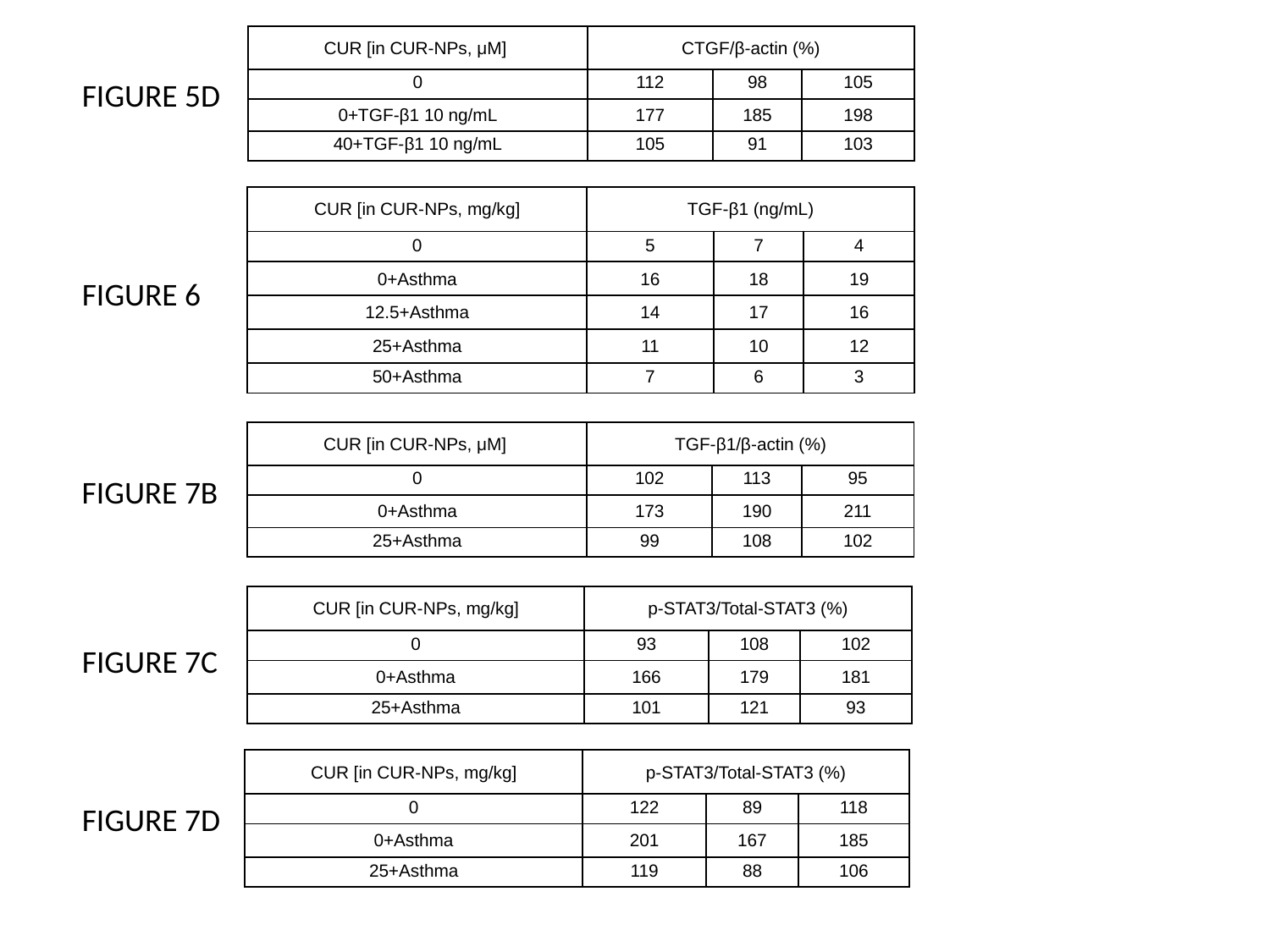

| CUR [in CUR-NPs, μM] | CTGF/β-actin (%) | | |
| --- | --- | --- | --- |
| 0 | 112 | 98 | 105 |
| 0+TGF-β1 10 ng/mL | 177 | 185 | 198 |
| 40+TGF-β1 10 ng/mL | 105 | 91 | 103 |
FIGURE 5D
| CUR [in CUR-NPs, mg/kg] | TGF-β1 (ng/mL) | | |
| --- | --- | --- | --- |
| 0 | 5 | 7 | 4 |
| 0+Asthma | 16 | 18 | 19 |
| 12.5+Asthma | 14 | 17 | 16 |
| 25+Asthma | 11 | 10 | 12 |
| 50+Asthma | 7 | 6 | 3 |
FIGURE 6
| CUR [in CUR-NPs, μM] | TGF-β1/β-actin (%) | | |
| --- | --- | --- | --- |
| 0 | 102 | 113 | 95 |
| 0+Asthma | 173 | 190 | 211 |
| 25+Asthma | 99 | 108 | 102 |
FIGURE 7B
| CUR [in CUR-NPs, mg/kg] | p-STAT3/Total-STAT3 (%) | | |
| --- | --- | --- | --- |
| 0 | 93 | 108 | 102 |
| 0+Asthma | 166 | 179 | 181 |
| 25+Asthma | 101 | 121 | 93 |
FIGURE 7C
| CUR [in CUR-NPs, mg/kg] | p-STAT3/Total-STAT3 (%) | | |
| --- | --- | --- | --- |
| 0 | 122 | 89 | 118 |
| 0+Asthma | 201 | 167 | 185 |
| 25+Asthma | 119 | 88 | 106 |
FIGURE 7D

## Slide 5
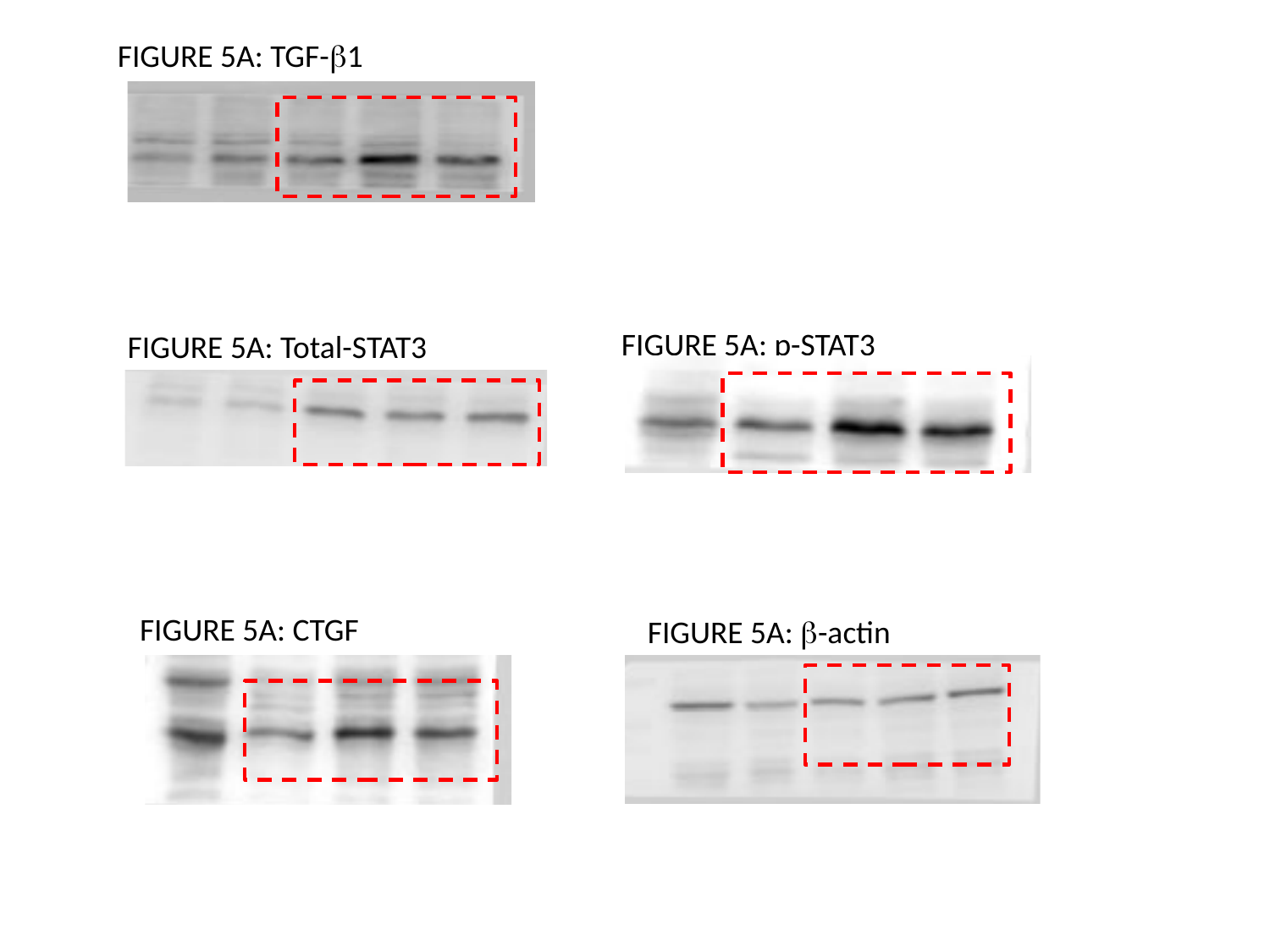

FIGURE 5A: TGF-b1
FIGURE 5A: p-STAT3
FIGURE 5A: Total-STAT3
FIGURE 5A: CTGF
FIGURE 5A: b-actin

## Slide 6
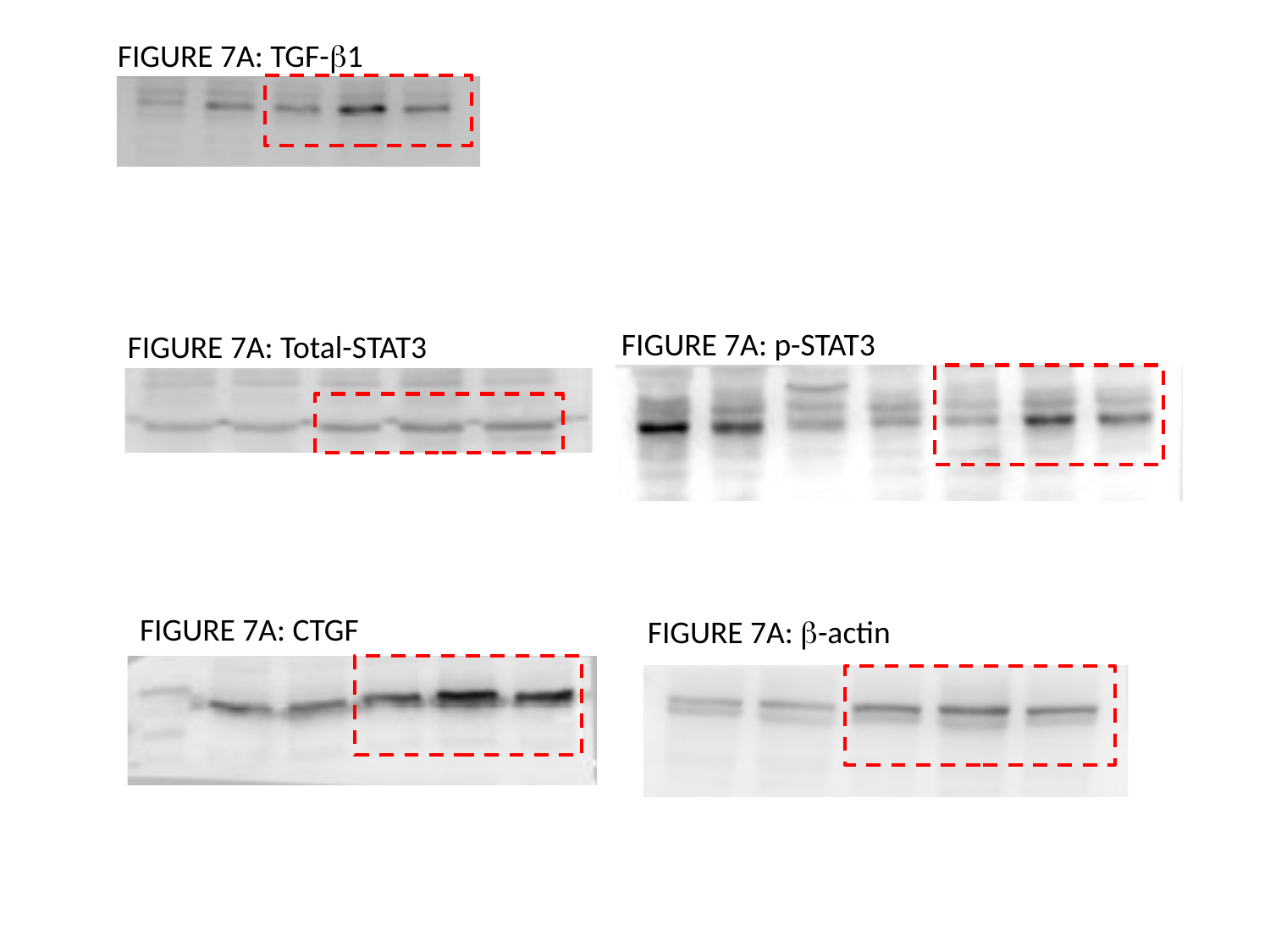

FIGURE 7A: TGF-b1
FIGURE 7A: p-STAT3
FIGURE 7A: Total-STAT3
FIGURE 7A: CTGF
FIGURE 7A: b-actin
